# Redescription of *Takahashia citricola* Kuwana, 1909, and its transfer to the genus *Pulvinaria* Targioni Tozzetti (Coccoidea, Coccidae)

**DOI:** 10.3897/zookeys.217.3405

**Published:** 2012-08-28

**Authors:** Hirotaka Tanaka

**Affiliations:** 1 Tottori Prefectural Museum, 2–124 Higashi-machi, Tottori City, 680–0011 Japan

**Keywords:** *Takahashia citricola*, *Pulvinaria citricola*, resurrected state, soft scale, Coccoidae

## Abstract

The Japanese soft scale*Takahashia citricola* Kuwana, 1909 is redescribed and transferred to the genus *Pulvinaria*Targioni Tozzettias *Pulvinaria citricola* (Kuwana, 1909), **comb. n.**(Coccoidea: Coccidae). *Pulvinaria gamazumii* Kanda, 1960 is synonymized with *Pulvinaria citricola* **comb. n.** and *Pulvinaria nipponica* Lindinger, 1933, is resurrected as the replacement name for *Pulvinaria citricola* Kuwana, 1914 (nec Kuwana, 1909). The adult female of *Pulvinaria citricola* (Kuwana, 1909) is redescribed and illustrated.

## Introduction

Soft scale insects belong to the family Coccidae which is of the third largest family in the superfamily Coccoidea (Hemiptera: Sternorrhyncha), with more than 1100 species described until now (Ben-Dov 2012a). Like members of other families of the Coccoidea, species in the family Coccidae are known for their peculiar morphology compared with other “normal” insects, and are also economically important as plant pests. Like aphids (superfamily Aphidoidea), they suck plant sap and host plants affected by them will become debilitated or die if their populations become too high. Their “honeydew” can cause sooty molds on plant surfaces and cause indirect damage to the plant by reducing their photosynthetic activity. Furthermore, some soft scale insects are known as vectors of serious plant diseases such as Grapevine leafroll (Belli et al. 1994, Fortusini et al. 1997, Hommay et al. 2008). Thus, control of soft scale insects and the study of their taxonomy are very important issues for worldwide crop production, especially for fruit crops.

In Japan, 72 species of soft scales (Coccidae) in 21 genera have been recorded (Ben-Dov 2012b, 2012c), but Japanese genera and species of Coccidae are still in need of study. An example is the Japanese species *Takahashia citricola* Kuwana, 1909, for which the adult female has a discal seta on each anal plate – a feature considered diagnostic for members of the genus *Saissetia* Déplanche. Probably for this reason, *Takahashia citricola* has been treated as *Saissetia citricola* (Kuwana 1909) (Takahashi and Tachikawa 1956, Ben-Dov 1993). However detailed study of the morphology of *Takahashia citricola* (presented below) shows that it should be transferred to the genus *Pulvinaria* Targioni Tozzetti. However the name ‘citricola’ is in use in *Pulvinaria* for a different species, *Pulvinaria citricola* Kuwana, 1914 (nec Kuwana, 1909), which thus becomes a junior homonym. *Pulvinaria nipponica* Lindinger, 1933, is resurrected here as the replacement name for *Pulvinaria citricola* Kuwana, 1914. The adult female of *Pulvinaria citricola* (Kuwana, 1909) is redescribed and illustrated and some taxonomic features of the species are discussed.

The genus *Pulvinaria* have been split in the past into other genera such as *Acanthopulvinaria* (Borchsenius 1952), *Anapulvinaria* (Borchsenius 1952), *Chloropulvinaria* (Borchsenius 1952), *Eupulvinaria* (Borchsenius 1953), *Megapulvinaria* (Yang 1982), *Neopulvinaria* (Hadzibejli 1955), *Pulvinella* (Hempel 1900), *Pulvinariella* (Borchsenius 1953), *Pulvinarisca* (Borchsenus 1953), and *Saccharipulvinaria* (Tao et al. 1983). However, the latter genera have been rarely accepted by current taxonomists (e.g. Williams and Watson 1990) and taxonomy of the tribe Pulvinariini (*Pulvinaria* and related genera) is in great need of further study. Under these circumstances, the genus *Pulvinaria* is here treated in the broad sense.

## Materials and methods

The scale insect samples studied were collected by several Japanese coccidologists (S. Kanda, S. Kawai and R. Takahashi) plus newly collected specimens. The slide-mounting method for the newly collected specimens followed that of Kawai (1980). The morphology of the slide-mounted specimens was examined under a phase-contrast light microscope (Olympus BH-2 PH). Terminology follows Hodgson (1997). Specimens examined in this study are deposited in the Osaka Museum of Natural History, Osaka (**OMNH**), The Laboratory of Systematic Entomology, Faculty of Agriculture, Hokkaido University, Sapporo **(SEHU),** and at the Kawai scale insect collection deposited at Tokyo University of Agriculture, Tokyo (**TUA**).

## Taxonomy

### Pulvinaria citricola

(Kuwana, 1909) comb. n.

http://species-id.net/wiki/Pulvinaria_citricola

Figs 1 2

Takahashia citricola Kuwana 1909: 153; Kuwana 1917: 53.
Saissetia citricola : Takahashi and Tachikawa 1956: 7; Kawai 1972: 17; Kawai 1980: 157; Tang 1991: 216; Ben-Dov 1993: 304; Kozár et al. 1998: 152.
Pulvinaria gamazumii Kanda 1960: 119. syn. n.
Parasaissetia citricola : Yang 1982: 178.

#### Material examined

(measured specimens are indicated by asterisks). 4 females*, **Japan:** Ôsaka [Osaka](34°33'N, 135°35'E - 34°46'N, 135°23'E), 5.v.1954, R. Takahashi coll., host: *Cinnamomum camphora* (deposited in SEHU). 1 female*, **Japan:** Aomori, Towada (40°25'N, 141°20'E - 40°39'N, 140°50'E), no date indicated, S. Kanda coll., host: *Viburnum wrightii* (deposited in OMNH; a syntype of *Pulvinaria ganazumii*). 5 females*, **Japan:** Tiba [Chiba], Matudo [Matsudo], Kamihongô [Kamiongo] (35°47'N, 139°55'E), 23.iv.2005, H. Tanaka coll., host: *Ilex integra* (deposited in TUA). 4 females, **Japan:** Tôkyô [Tokyo], Meguro (35°35'N, 139°39'E - 35°39'N, 139°44'E), 26.iv.1972, S. Kawai coll., host: *Illicium anisatum* (deposited in TUA); 1 female, Japan: Tôkyô [Tokyo], Hutyû [Fuchu], Saiwai-tyô [Saiwai-cho](35°41'N, 139°29'E), 10.v.1974, S. Kawai coll., host: *Laurus nobilis* (deposited in TUA).

#### Species diagnosis

(based on adult female). Body nearly round, broadest across thorax and anterior abdomen. Body strongly lifted by ventral ovisac. Anal plates posteriorly elongate; each plate with posterior margin about two times as long as anterior margin, with 3 fine apical setae and a well-developed discal seta. Three types of ventral tubular ducts present; smallest type forming complete broad submarginal band.

#### Description.

**Figure 1.**
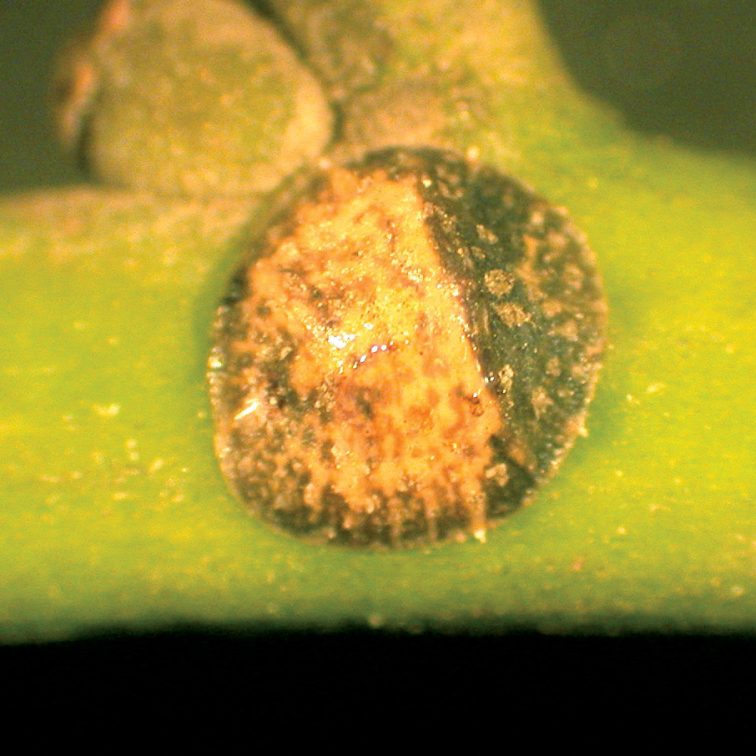
*Pulvinaria citricola* (Kuwana, 1909), a pre-oviposition adult female on *Ilex integra*.

**Living appearance**. Body nearly round, broadest across thorax and anterior abdomen. Prior to oviposition, dorsum reddish light brown posteriorly and dark brown anteriorly (Fig. 1), changing to reddish brown with a dark brown marginal ring at full-maturity. Mature adult females produce an ovisac mainly from beneath abdomen. No wax present on dorsum at oviposition. Post-oviposition females sclerotised and not shrivelled. Ovisac white, very convex, nearly hemispherical. Body strongly lifted posteriorly by ovisac.

**Slide-mounted material (measured individuals: n = 10).**Body elongate-oval, 2.9–4.0 mm long, 2.9–4.0 mm wide, margin with a shallow indentation at each stigmatic cleft; anal cleft 1/5–1/6 body length.

*Dorsum*. Derm membranous when young. Dermal areolations well developed. Dorsal setae spiniform, 7–12 mm long, frequent, scattered over entire dorsum, each with a well-developed basal socket. Preopercular pores each ring-like, conspicuous, 3.5–7.0 mm wide, consisting of a compact group of 2–11 pores anterior to anal plates. Dorsal tubular ducts absent. Dorsal microductules frequent throughout. Dorsal tubercles absent. Anal plates posteriorly elongated; each plate with posterior margin about two times as long as anterior margin, each with 3 fine apical setae and a discal seta; length of plates 214–249 mm; width of single plate 85–122 mm; each plate with well-developed supporting bars. Ano-genital fold with 2 pairs of setae along anterior margin and 1 or 2 pairs laterally. Anal ring with 6–8 setae (mostly 8). Eyespot present near margin

*Margin*.Marginal setae spinose, each with a simple pointed apex and a well-developed basal socket; length highly variable, each seta 20–107 mm long, with 10–13 setae present on each side between stigmatic clefts. Stigmatic clefts shallow, each with 0–3 (mostly 3) stigmatic spines in each cleft, longest spine 92–134 µm long, about 3–4 times as long as lateral spines.

*Venter*.Derm membranous. Pregenital disc-pores each with 8–11 loculi (mainly 10), present around genital opening and on mediolateral areas of all abdominal segments and metathorax; a small group also present laterad of each coxa. Spiracular disc-pores each with 5 loculi, present between margin and each spiracle in band 1–5 pores wide; anterior bands with 30–63 pores and posterior bands with 45–80 pores. Ventral microducts evenly distributed throughout venter. Three types of ventral tubular ducts present: type I with well-developed terminal gland and a stout inner ductule, present medially on head and thorax; type II rather narrower with a long, much thinner inner ductule, shallow cup-shaped invagination, and a well-developed terminal gland, sparse medially on posterior abdominal segments and also in a submarginal band with ducts of type III; type III ducts similar to type II, but with a short, filamentous inner ductule and very small terminal gland, mainly present in a broad submarginal band. Ventral setae: 3 pairs of long pregenital setae and 3–6 pairs of long setae between antennae; other setae short and fine. Spiracles as usual for family; width of each peritreme: anterior 47–60 mm, posterior 58–70 mm. Legs well developed; each with tibio-tarsal articulation and articulatory sclerosis; all claws without denticle; both claw digitules rather broad and slightly shorter than thin tarsal digitules; trochanter + femur 240–306 mm, tibia 162–198 mm, and tarsus 92–118 mm. Antennae each with 7 or 8 segments (mostly 8); total length 342–412 mm. Labium approximately 47–118 mm wide.

#### Host plants in Japan.

Aquifoliaceae: *Ilex integra* (Kawai 1980); *Ilex chinensis* (Kawai 1980). Araliaceae: *Fatsia japonica* (Takahashi and Tachikawa 1956, Kawai 1980). Adoxaceae: *Viburnum odoratissimum* (Takahashi and Tachikawa 1956, Kawai 1980); *Viburnum wrightii* (Kanda1960).Ebenaceae: *Diospyros kaki* (Takahashi and Tachikawa 1956). Sapindaceae: *Aesculus turbinata* (Kawai 1980). Schisandraceae: *Illicium anisatum* (Kawai 1980). Lauraceae: *Cinnamomum camphora* (Takahashi and Tachikawa 1956, Kawai 1980); *Laurus nobilis* (Takahashi and Tachikawa 1956, Kawai 1980); *Machilus thunbergii* (Kawai 1980); *Lindera erythrocarpa* (Kawai 1980). Magnoliaceae: *Magnolia kobus* (Takahashi and Tachikawa 1956, Kawai 1980). Pentaphylacaceae: *Eurya japonica* (Kawai 1980); *Ternstroemia gymnanthera* (Kawai 1980). Pittosporaceae: *Pittosporum tobira* (Kawai 1980). Rosaceae: *Pyrus pyrifolia* (Takahashi and Tachikawa 1956, Kawai 1980). Rubiaceae: *Gardenia jasminoides* (Kawai 1980). Rutaceae: *Citrus* sp. (Kuwana 1909). Styracaceae: *Styrax obassia* (Kawai 1980). Theaceae: *Camellia sinensis* (Kawai 1980).

#### Distribution.

Thisspecies occurs widely across central and northern parts of Japan from Honsyû [Honsyu], Kyusyû [Kyushu] and Sikoku [Shikoku] districts.

#### Notes.

A good photograph of ovisac-producing adult females is presented in Kawai (1980). Kuwana’s type material of this species could not be found in the Kuwana collection of the National Institute of Agricultural Environmental Sciences, Tukuba [Tsukuba] and is presumed lost.

### Pulvinaria nipponica

Lindinger, 1933 stat. res.

Pulvinaria citricola  Kuwana 1914: 3; Kuwana 1917: 35; Steinweden 1946: 5; Takahashi 1955: 151; Takahashi and Tachikawa 1956: 6; Williams and Kosztarab 1972: 125; Kawai 1972: 15; Kawai 1980: 154; Gill 1988: 85; Tang 1991: 234; Tao et al. 1990: 64; Ben-Dov 1993: 254.
Pulvinaria nipponica Lindinger, 1933: 46 [Replacement name].
Eupulvinaria citricola : Borchsenius 1953: 288.

#### Notes.

As explained in the introduction, the transfer of *Takahashia citricola* Kuwana, 1909, to *Pulvinaria*necessitates a replacement name for the junior homonym *Pulvinaria citricola* Kuwana, 1914. A previously unjustified replacement name *Pulvinaria nipponica* was proposed by Lindinger (1933) for *Pulvinaria citricola* Kuwana, 1914, and this earlier name satisfies the provision of article 11 and the requirement of the article 13 (13.1.3.) of The International Code of Zoological Nomenclature (ICZN 1999). Thus, its’ status is resurrected as the replacement name for *Pulvinaria citricola* Kuwana, 1914 (nec Kuwana, 1909) in accordance to articles 23 (23.3.5.) and 60 (60.1. and 60.2.) of the ICZN.

## Discussion

*Pulvinaria citricola* comb. nov. resembles the Japanese species *Pulvinaria nipponica* stat. res., *Pulvinaria kuwacola* Kuwana and *Pulvinaria photiniae* Kuwana in the distribution of type III ventral tubular ducts and the shape of marginal setae. However, it is easily distinguishable from those three species in having a well-developed discal seta on each anal plate and by the shape of the anal plates which are posteriorly elongated (*Pulvinaria nipponica*, *Pulvinaria kuwacola* and *Pulvinaria photiniae* lack discal setae and their anal plates are together quadrate and not elongated posteriorly).

Most soft scales in the genus *Saissetia* (Coccidae: Coccinae: Saissetini) also have a discal setae on the anal plates and this is considered a diagnostic feature of the genus (Williams and Watson 1990, Hodgson 1994). Takahashi and Tachikawa (1956) probably considered this feature when they transferred *Takahashia citricola* to the genus *Saissetia*, though they did not clearly express the reasons for the transfer. However, the distributional pattern of the ventral tubular ducts of *Pulvinaria citricola* (Kuwana, 1909) greatly differs from most species of *Saissetia* including the type species of the genus and is similar to that of many *Pulvinaria* species. Furthermore, ovipositing adult females of *Pulvinaria citricola* (Kuwana, 1909) produce a well-developed ovisac (see Kawai 1980), which is the most important diagnostic feature of the genus *Pulvinaria* and all of the related genera in the tribe Pulvinariini.

**Figure 2.**
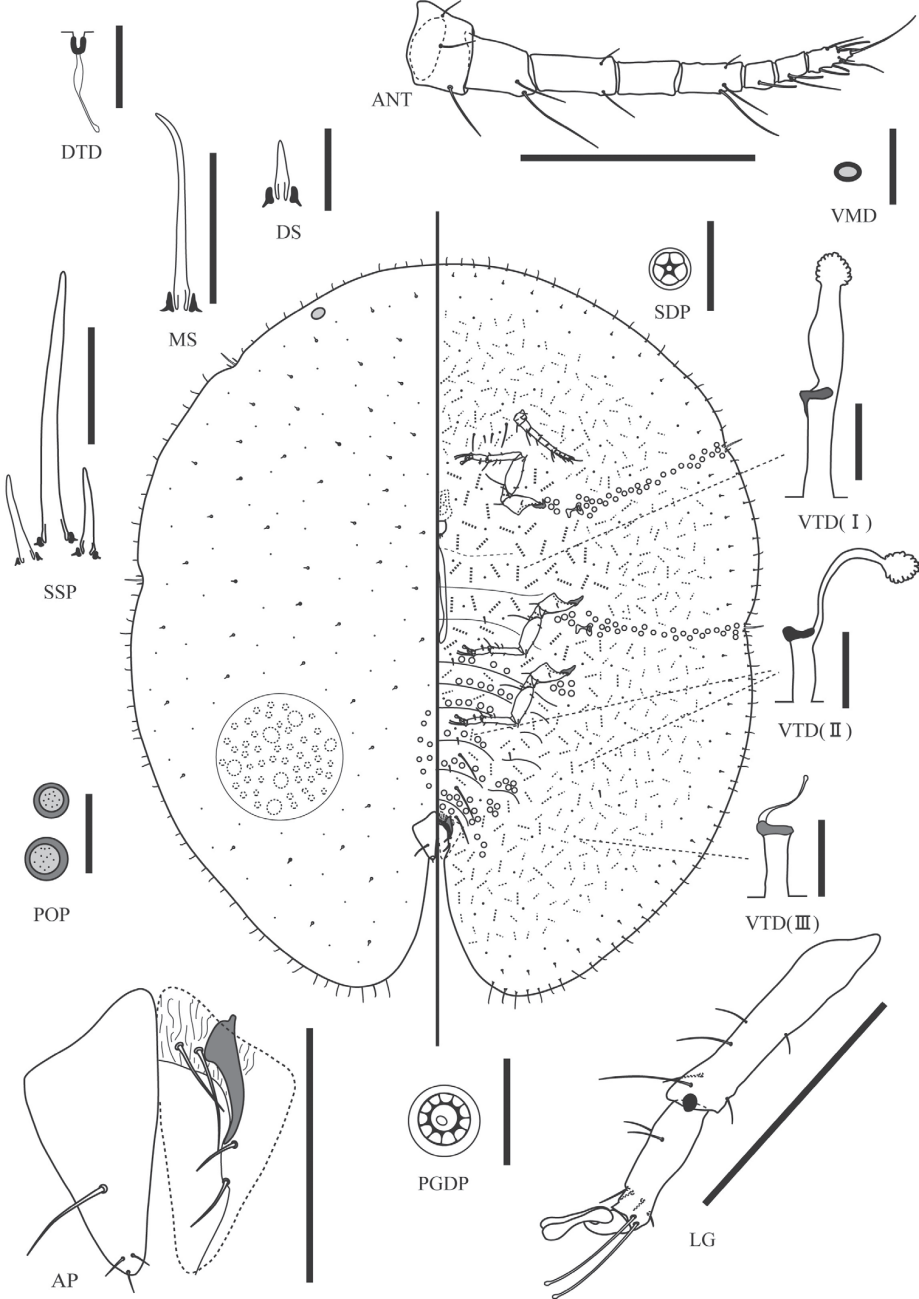
*Pulvinaria citricola* (Kuwana, 1909), adult female. ANT, antenna; AP, anal plates; DMD, dorsal microductule; DS, dorsal seta; LG, leg; MS, marginal setae; PGDP, pregenital disc pore; POP, preopercular pores; SDP, spiracular disc pore; SSP, stigmatic spines; VMD, ventral microduct; VTD, ventral tubular ducts of types I–III. Scales: 200 mm for ANT, AP, LG; 50 mm for MS, SSP; 10 mm for others.

## Supplementary Material

XML Treatment for Pulvinaria citricola

XML Treatment for Pulvinaria nipponica

## References

[B1] Belli G Fortusini A Casati P Belli L Bianco PA Prati S (1994) Transmission of a grapevine leafroll associated closterovirus by the scale insect *Pulvinaria vitis* L. Rivista di Patologia Vegetale 4: 105-108

[B2] Ben-Dov Y (1993) A Systematic Catalogue of the Soft Scale Insects of the World (Homoptera: Coccoidea: Coccidae) with data on geographical distribution, host plants, biology and economic importance. Flora and Fauna Handbook, No. 9. Sandhill Crane Press, Gainesville, 536 pp.

[B3] Ben-Dov Y (2012a) ScaleNet, Family Coccidae. http://www.sel.barc.usda.gov/scalecgi/chklist.exe?Family=Coccidae&genus= [accessed at 9 July 2012]

[B4] Ben-Dov Y (2012b) ScaleNet, Scales in a Country Query Results. http://www.sel.barc.usda.gov/scalecgi/region.exe?region=P&family=Coccidae&country=JPN&genus=&intro=A&detail=No&subunit=&regname=Palaearctic&ctryname=&action=Submit+Query&querytype=Country+Query [accessed at 18 July 2012]

[B5] Ben-Dov Y (2012c) ScaleNet, Scales in a Country Query Results. http://www.sel.barc.usda.gov/scalecgi/region.exe?region=O&family=Coccidae&country=RYU&genus=&intro=A&detail=No&subunit=&regname=Oriental&ctryname=&action=Submit+Query&querytype=Country+Query [accessed at 18 July 2012]

[B6] Borchsenius NS (1952) [New genera and species of soft scales of the family Coccidae (=Lecaniidae) of the USSR fauna and adjacent countries (Insecta, Homoptera, Coccoidea)]. Trudy Zoologicheskogo Instituta Akademii Nauk SSSR 12: 269-316 [In Russian]

[B7] Borchsenius NS (1953) [New genera and species of scale insects of the family Coccidae (Homoptera, Coccoidea)]. Entomologicheskoe Obozrenye 33: 281-290 [In Russian]

[B8] Fortusini A Scattini G Prati S Cinquanta S Belli G (1997) Transmission of grapevine leafroll virus 1 (GLRaV-1) and grapevine virus A (GVA) by scale insects. Proceedings of 12th Meeting of ICVG, Lisbon (Portugal), September/October 1997, 121–122

[B9] Gill RJ (1988) The Scale Insects of California: Part 1. The Soft Scales (Homoptera: Coccoidea: Coccidae). California Dept. of Food and Agriculture, Sacramento, CA, 132 pp.

[B10] Hadzibejli ZK (1955) [New genus and species of the soft scales family Lecaniidae (Homoptera, Coccoidea) from Georgia]. Entomologicheskoe Obozrenye 34: 231–239 [In Russian]

[B11] Hempel A (1900) As coccidas Brasileiras. Revista do Museu Paulista. São Paulo 4: 365-537 [In Portuguese]

[B12] Hodgson CJ (1994) The Scale Insect Family Coccidae: an identification manual to genera. CAB International, Wallingford, Oxon, 639 pp.

[B13] Hodgson CJ (1997) Taxonomic characters–adult female. In: Ben-Dov Y Hodgson CJ (Eds). Soft Scale Insects–Their Biology, Natural Enemies and Control.Elsevier, Amsterdam and New York: 111-137 doi: 10.1016/S1572-4379(97)80047-4

[B14] Hommay G Komar V Lemaire O Herrbach E (2008) Grapevine virus A transmission by larvae of *Parthenolecanium corni*. European Journal of Plant Pathology 121: 185-188 doi: 10.1007/s10658-007-9244-3

[B15] ICZN (1999) International Code of Zoological Nomenclature, Fourth edition. International Trust for Zoological Nomenclature

[B16] Kanda S (1960) Descriptions of the Coccidae from Japan (Homoptera). Kontyû 28: 116-123

[B17] Kawai S (1972) [Diagnostic notes and biology of the coccid species occurring on cultivated or wild trees and shrubs in Japan (Homoptera: Coccoidea)]. Bulletin of the Tokyo-to Agricultural Experiment Station 6: 1-54 [In Japanese]

[B18] Kawai S (1980) [Scale Insects of Japan in Colors]. Zenkoku Nôson Kyouiku Kyoukai, Tokyo, 455 pp.[In Japanese]

[B19] Kozár F Konczné Benedicty Z Drozdják J (1998) Coccidae. In: Kozár F (Ed). Catalogue of Palaearctic Coccoidea.Plant Protection Institute, Hungarian Academy of Sciences, Budapest: 41-164

[B20] Kuwana SI (1909) Coccidae of Japan (III). First supplemental list of Japanese Coccidae, or scale insects, with description of eight new species. Journal of the New York Entomological Society 17: 150-158

[B21] Kuwana SI (1914) Coccidae of Japan, V. Journal of Entomology and Zoology 6: 1-8

[B22] Kuwana SI (1917) [Coccidae of Japan, vol II.]. Nishigahara Sousyo Kankoukai, Tokyo, 157 pp.

[B23] Lindinger L (1933) Beiträge zur Kenntnis der Schildläuse (Hemipt. – Homopt., Coccid.). Entomologischer Anzeiger 13: 77-166

[B24] Steinweden JB (1946) The identity of certain common American species of *Pulvinaria* (Homoptera: Coccoidea: Coccidae). Microentomology 11: 1–28

[B25] Takahashi R (1955) *Pulvinaria* of Japan (Coccidae, Homoptera). Kontyû 23: 148-154

[B26] Takahashi R Tachikawa T (1956) Scale insects of Shikoku (Homoptera: Coccoidea). Transactions of the Shikoku Entomological Society 5: 1-17

[B27] Tang FT (1991) [The Coccidae of China]. Shanxi United Universities Press, Taiyuan, 377 pp. [In Chinese]

[B28] Tao CCC Wong CY Chang YC (1983) Monograph of Coccidae of Taiwan, Republic of China (Homoptera: Coccoidea). Journal of Taiwan Museum 36: 57-107

[B29] Williams ML Kosztarab M (1972) Morphology and systematics of the Coccidae of Virginia with notes on their biology (Homoptera: Coccoidea). Research Division Bulletin, Virginia Polytechnic Institute and State University 74: 1–215

[B30] Williams DJ Watson GW (1990) The Scale Insects of the Tropical South Pacific Region. Pt. 3: The soft scales (Coccidae) and other families. CAB International Institute of Entomology, London, 267 pp.

[B31] Yang PL (1982) [General classification of scale insects in China]. Shanghai Science and Technology, Shanghai, 425 pp. [In Chinese]

